# Predictive modelling using pathway scores: robustness and significance of pathway collections

**DOI:** 10.1186/s12859-019-3163-0

**Published:** 2019-11-04

**Authors:** Marcelo P. Segura-Lepe, Hector C. Keun, Timothy M. D. Ebbels

**Affiliations:** 1 Computational and Systems Medicine, Department of Surgery and Cancer, Sir Alexander Fleming building, Imperial College, London, SW1 2AZ UK; 2 Division of Cancer, Department of Surgery and Cancer, Imperial College London, Hammersmith Hospital Campus, W12 0NN, London, UK

**Keywords:** Pathways, Robustness, Predictive modelling

## Abstract

**Background:**

Transcriptomic data is often used to build statistical models which are predictive of a given phenotype, such as disease status. Genes work together in pathways and it is widely thought that pathway representations will be more robust to noise in the gene expression levels. We aimed to test this hypothesis by constructing models based on either genes alone, or based on sample specific scores for each pathway, thus transforming the data to a ‘pathway space’. We progressively degraded the raw data by addition of noise and examined the ability of the models to maintain predictivity.

**Results:**

Models in the pathway space indeed had higher predictive robustness than models in the gene space. This result was independent of the workflow, parameters, classifier and data set used. Surprisingly, randomised pathway mappings produced models of similar accuracy and robustness to true mappings, suggesting that the success of pathway space models is not conferred by the specific definitions of the pathway. Instead, predictive models built on the true pathway mappings led to prediction rules with fewer influential pathways than those built on randomised pathways. The extent of this effect was used to differentiate pathway collections coming from a variety of widely used pathway databases.

**Conclusions:**

Prediction models based on pathway scores are more robust to degradation of gene expression information than the equivalent models based on ungrouped genes. While models based on true pathway scores are not more robust or accurate than those based on randomised pathways, true pathways produced simpler prediction rules, emphasizing a smaller number of pathways.

## Background

A common objective in modern biological investigations is to use transcriptomic or other ‘omics’ data to develop statistical models predictive of a given phenotype, such as disease status or prognosis. The main goal of such studies is often to identify groups of genes, or signatures, which are associated with the desired outcome. However, data from all omics technologies are subject to a wide variety of technical noise and biological variation, which will degrade the performance of these models and limit the fidelity with which predictive signatures can be identified.

In nature, genes and other biomolecules function together in pathways and it was proposed early in post-genomic science that grouping genes into pathways could circumvent the difficulties with noise suffered by gene focused analysis [1, 2]. While it is widely thought that such pathway analyses are robust to noise in the data, the existing approaches have attempted to demonstrate robustness only indirectly, through effects on maximal attainable predictive accuracy [3, 4], the consistency of selected features across data sets and methods [3–9], or through biological justification in case studies [10–13]. In contrast, here we focus exclusively on ‘predictive robustness’ understood as the model’s capacity to maintain predictive accuracy in the face of uncontrolled variation, and not referring to other biological or statistical notions of robustness. Note that a high robustness by our definition does not necessarily imply an extremely accurate predictive model; it merely requires the accuracy to be maintained above chance levels, as the level of irrelevant variation or noise in the data increases. Also note that the uncontrolled variation may result from a variety of sources, including both technical noise and biological (inter-subject) variation. The latter often dominates the total variance in typical datasets, but it may not be useful for predicting the phenotype of interest. Thus it can be seen as a kind of biological ‘noise’ against which the model must remain robust. This is the scenario which we address in the current work.

Predictive models are usually built using gene expression levels as variables, hence such models are built in the ‘gene space’. However, the idea of predictive modelling can be combined with pathway analysis, so that predictive models are built instead in a ‘pathway space’ [14] where each variable relates separately to the ‘activity’ of a pathway for each sample. The main hypothesis of this paper is that pathway based predictive models would show higher predictive robustness to noise in the raw data than those based on the individual gene expression levels. Our main aim is to test this hypothesis, by building predictive multivariate models in both gene and pathway space and examining their robustness to increasing degradation of the input data. We investigated the influence of different methods of introducing noise, and obtaining pathway scores, and whether the effects are replicated in two data sets.

Although we employ a type of pathway scoring, it is not our aim to introduce a new method for that purpose. The most common type of pathway analysis outputs a single score for each pathway (irrespective of the number of samples), which can be tested to determine pathways significantly associated with the outcome of interest [15]. Our pathway space modelling requires a score for each biological sample for each pathway, allowing the full data set to be modelled in the new pathway space. We employed a simple method based on principal components analysis (PCA) to do this, but many other approaches could be used e.g. [9, 16].

The coordinated action of genes in pathways naturally leads to the idea that pathways are ‘special’ collections of genes and thus models based on true pathways should be more predictive than those based on groups of unrelated genes. We therefore conjectured that performance of predictive models could be used to differentiate collections of real pathways from random collections of genes, thus addressing pathway significance. Surprisingly, we find that the predictive accuracy and robustness of models based on random gene sets can be similar to that of models based on pathways from databases. This counterintuitive result motivated us to examine other aspects of our models that could differentiate real and random pathway sets. We found that models based on true pathways are significantly simpler, in that they assign strong weights to fewer pathways, as compared to randomised versions of the same pathways. This yields an intrinsic “signature” to characterise different pathway collections such as those in existing databases.

## Results

### Pathway space representation

**Fig. 1:**
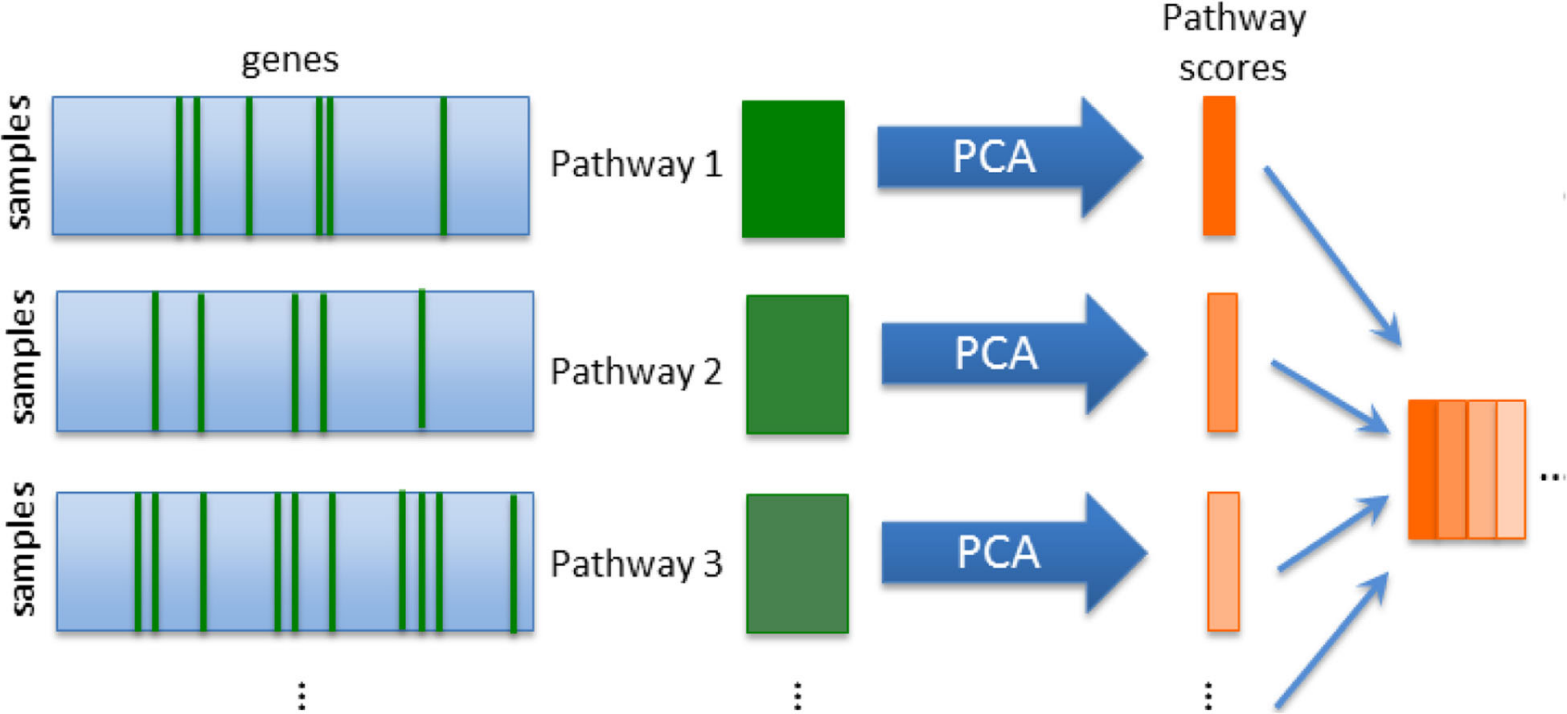
Workflow designed to produce a pathway space representation of a gene expression data set. The expression values for all *q*_*i*_ genes in the *i’*th pathway (green columns) are extracted from the original (*nxm)* expression matrix to form a new (*nxq*_*i*_) matrix. PCA is performed on this matrix and the score on the first component retained as the pathway score. This is repeated for each of the k pathways and the pathway scores assembled into a new ‘pathway space’ matrix of dimension (*nxk)* (orange)

In order to assess the contribution of pathway information to the robustness of predictive models we defined pathway scores that combined the expression of genes in each pathway using PCA. We calculated a one component PCA model for each pathway and compiled the scores into a new matrix that represents the samples in a “pathway space” (Fig. 1). Separate predictive models in both gene and pathway space were trained and cross validated using partial least squares – discriminant analysis (PLS-DA). The predictive ability of these models was calculated as the accuracy to assign a battery of compounds to one of three carcinogenic classes [17].

### Models in pathway space are more robust to noise than models in gene space

We examined the robustness of predictive models to degradation of the raw data. As more noise is added to the data, models whose accuracy declines slowly are deemed to be more robust than those whose accuracy declines quickly. Having predictive models both in pathway space and in the original gene space allowed us to study the contribution of pathway information to this type of robustness.

**Fig. 2:**
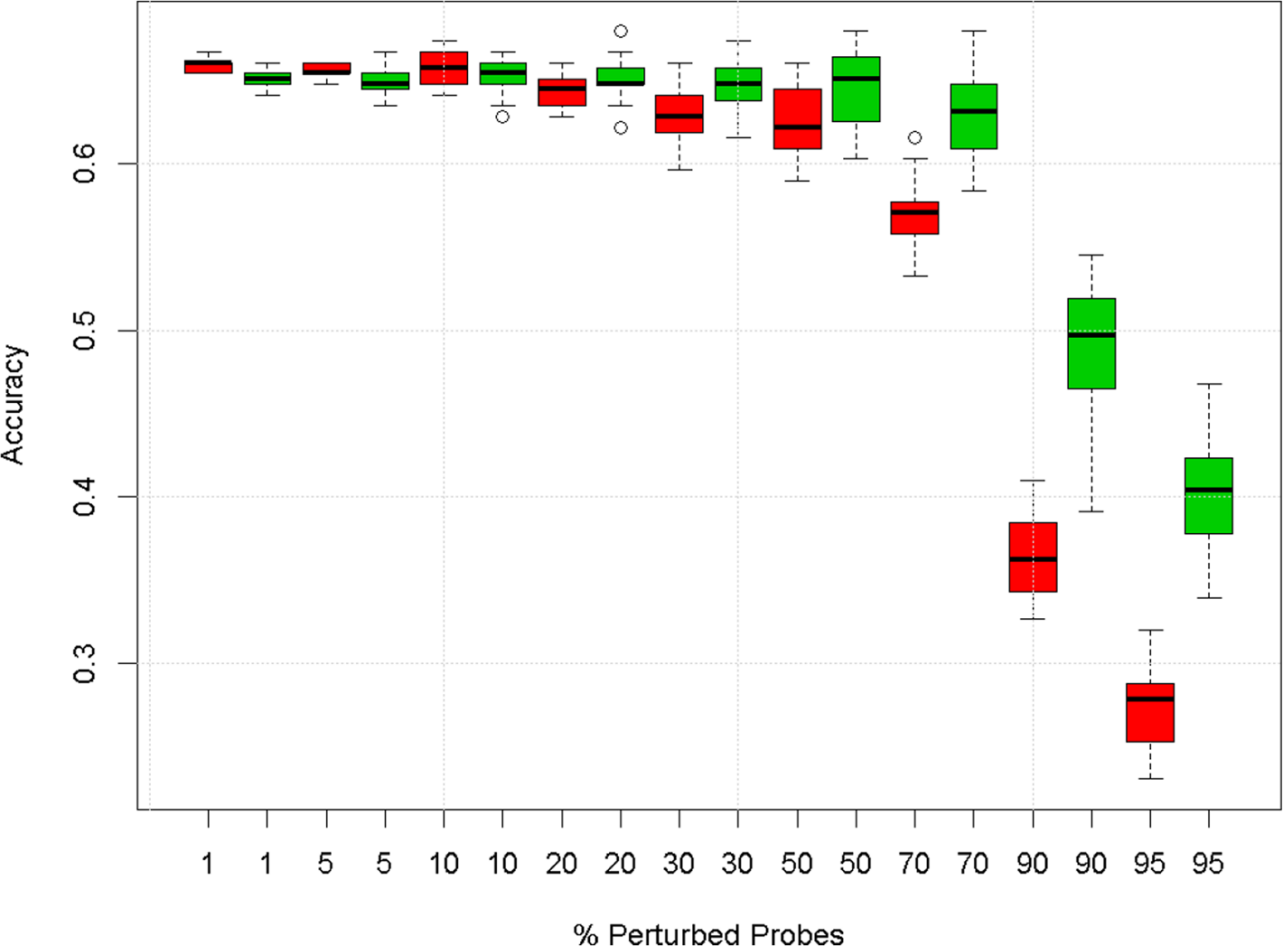
Degradation profiles from predictive models in gene (red) and pathway space (green) using the CG data set and the default workflow. Boxes represent inter-quartile ranges of accuracy for 20 realisations of the noise

The degradation profile of Fig. 2 shows that, while both gene space and pathway space models have similar predictive accuracy on non-degraded data, the accuracy of the gene level models decreases more quickly than those built in the pathway space as more noise is added to the data. For a more objective assessment of the predictive robustness, we calculated a “predictive robustness” statistic corresponding to the area under the degradation profile described by the medians and normalised to the accuracy of the model with unperturbed data (see Methods). The predictive robustness of pathway space models was significantly higher than that of gene space models (0.90 [0.89, 0.91] and 0.82 [0.81, 0.83] respectively, square brackets denote 90% confidence intervals.).

### Higher robustness of pathway models is independent of the workflow

**Fig. 3:**
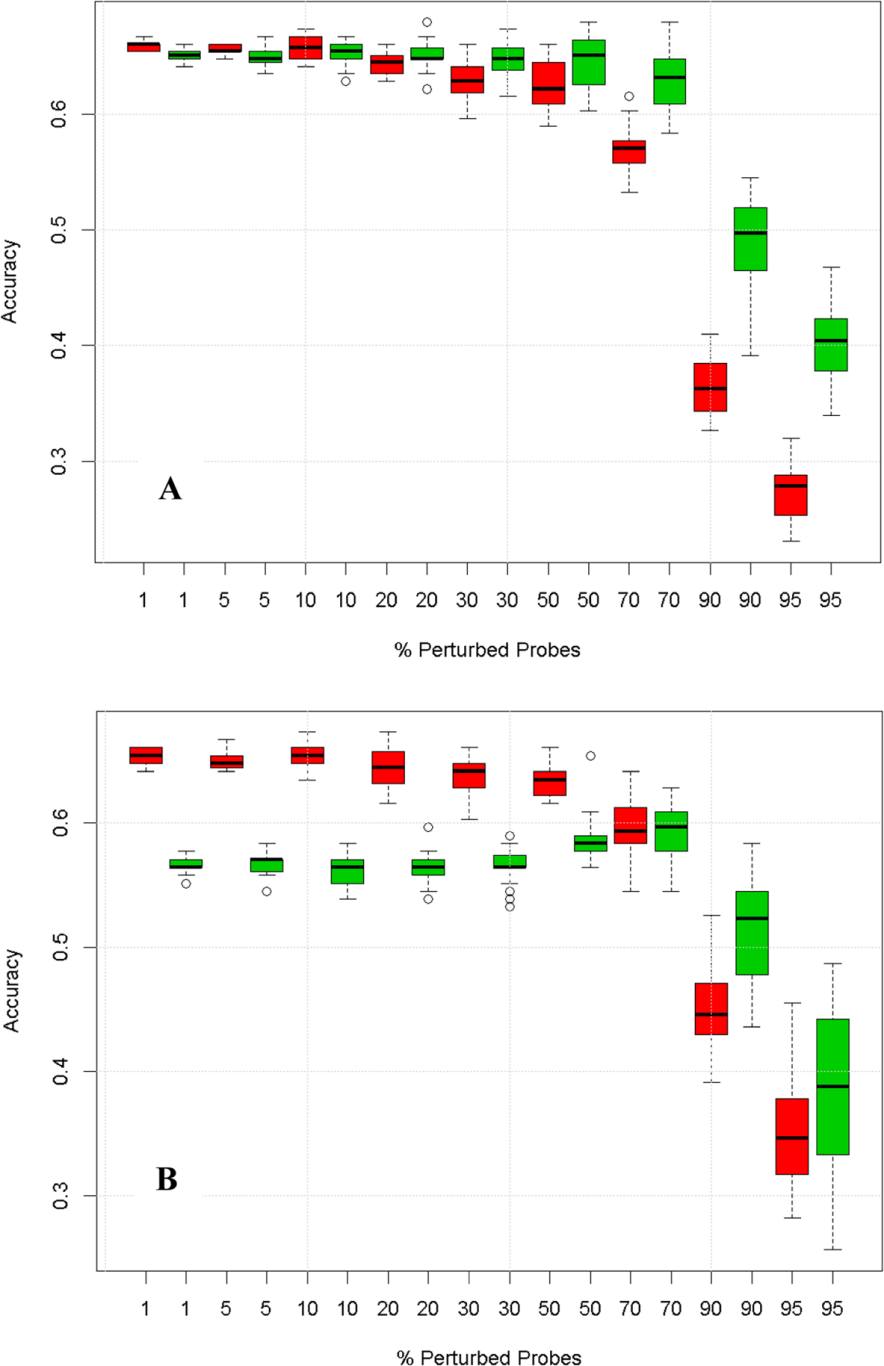
Degradation profiles for alternative workflows. **a** degradation by random permutation of samples. **b** kNN classification. Boxes represent inter-quartile ranges of accuracy from 20 realisations of the noise

To investigate the influence of different components of the workflow on the predictive robustness, we replaced steps of the default workflow by alternative methods, one at a time. Firstly, we replaced the default degradation method by random permutation of the sample order for the degraded genes (Fig. 3a), or by a “global noise” strategy to degrade in parallel the information from all genes (Additional file 1: Figure S1.1). The classification method was also replaced by k-nearest neighbours (kNN) (Fig. 3b) and an SVM (Additional file 1: Figure S1.3). The observation of higher robustness of the pathway space models as compared to the gene space models was unaffected by these modifications and similar results were obtained when three principal components were used to represent the pathway scores instead of one (Additional file 1: Figure S1.2A). For pathway scores based on mean expression, the local robustness of gene space models appeared higher at moderate levels of degradation, while pathway space models appeared more robust at high levels of degradation (Additional file 1: Figure S1.2B). The variability of accuracies of pathway space models was also increased. Use of single sample gene set enrichment analysis (ssGSEA) to generate pathway scores also had little influence (Additional file 1: Figure S.1.2C). Finally, we also explored the use of gene selection to build the gene space models, but again found similar results (Additional file 1: Figure S1.4).

**Fig. 4:**
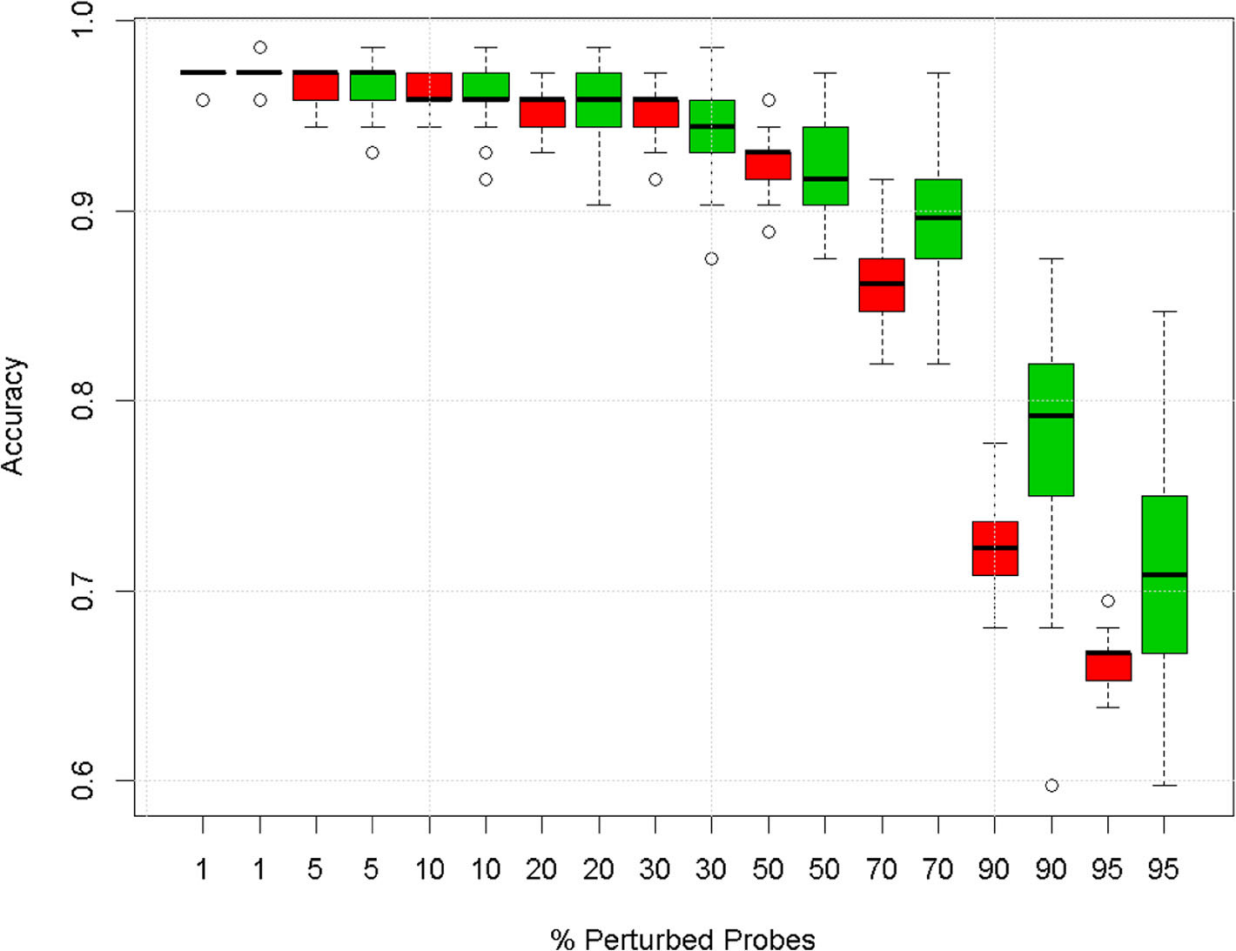
Degradation profile for the leukaemia data set. Boxes represent inter-quartile ranges of accuracy from twenty realisations

Aside from changes in the workflow, we also explored the robustness hypothesis on a second, unrelated, data set on leukaemia [18]. The degradation profiles in this dataset displayed similar predictive robustness as observed with the carcinoGENOMICS (CG) dataset (Fig. 4). This is notable considering that the two data sets have very different characteristics and were generated with different microarray platforms. The predictive robustness statistic confirmed the predictive robustness of pathway space models over gene space models over a broad range of changes to the data and workflow (Additional file 1: Table S1).

### Influence of specific pathway definition

**Fig. 5:**
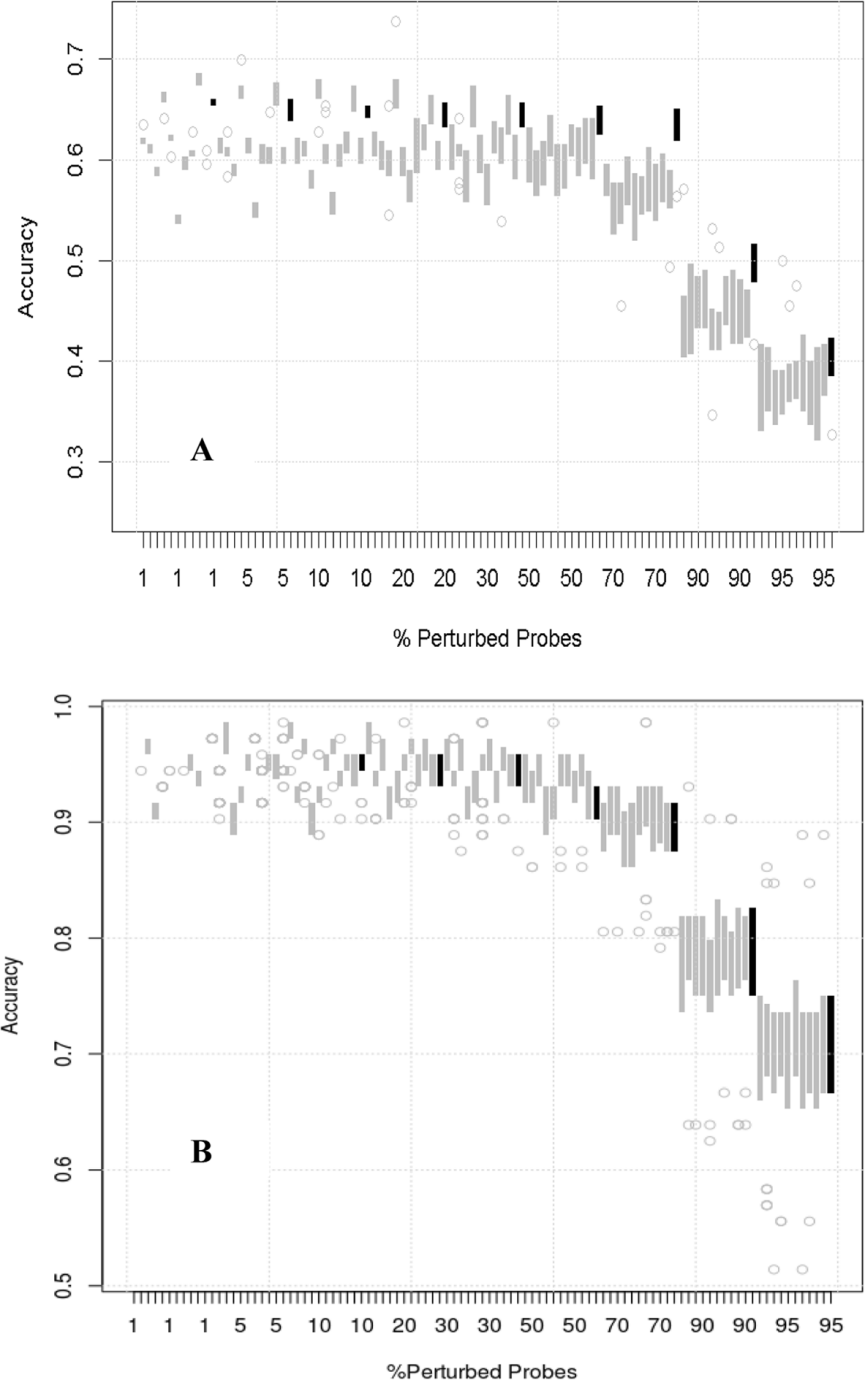
Predictive robustness in randomised pathway sets. Box plots of robustness in the true pathway set (black) and in ten fake pathway sets (grey) from the CG (**a**) and the leukaemia (**b**) data sets. All models were fit using the default workflow corresponding to Fig. 2

Having established the advantage of predictive models based on pathway scores, our next goal was to identify any underlying explanatory factors. Pathway databases reflect a corpus of existing biological knowledge and thus we hypothesised that the pathway composition, i.e. which genes are annotated to which pathway, has an impact on accuracy and robustness. We tested this hypothesis by creating databases of fake pathways which exactly replicated the size of the true pathways, as well as their overlap of shared genes. This approach is analogous to classical hypothesis testing in statistics, where a null distribution (here estimated by randomising pathway definitions) is used to assess the significance of an observed statistic (here accuracy and robustness). Surprisingly, the degradation profiles showed little or no difference between the true pathway set and the randomised versions (Fig. 5). We also used a second randomisation scheme that preserved only the sizes of the original pathways, which produced no qualitative changes (Additional file 1: Figure S2). This confirmed the observation that accuracy and predictive robustness are not properties of the gene composition of real pathways, suggesting that real pathway definitions are not “significant” according to accuracy and robustness.

### Models based on true pathways are simpler than those based on fake pathways

To explore the unexpected similarity of predictive robustness for true and fake pathway sets, we investigated the contribution of pathways across all predictive models in more detail. We hypothesized that for true pathway sets typically only a small number of pathways would be clearly influential for prediction, and conversely with randomised pathways, a larger number of pathways would be required, each one with a smaller contribution to the prediction. Note that in the remainder of this study, we only examine models based on the full, non-degraded data.

**Fig. 6:**
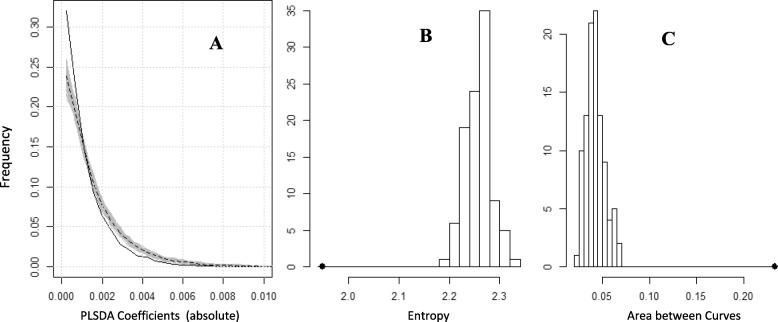
Distribution of pathway contributions to predictive models. **a** Distribution of absolute regression coefficients for PLS-DA models from 100 fake pathway sets (grey lines, median shown by black dashed line) or the true pathway set (black solid line). **b** Histogram of the entropies of the distributions in (A). **c** Histogram of the area between the distribution of coefficients for each fake pathway set and the median coefficient distribution (dashed black curve in A). The filled circles in B and C indicate the value for the true pathway set

In PLS-DA models, the regression coefficients indicate the contribution of pathways to the model and therefore we compared the distribution of PLS-DA coefficients from true and fake pathway sets (Fig. 6). For both true and fake pathway sets, the majority of the coefficients were low with a peak value close to zero (Fig. 6a). However, an excess of near-zero coefficients was observed for the true pathway set. Similarly, there was a lack of medium and high level coefficients in the models built from true pathways. We sought to capture these differences through measures of entropy (Fig. 6b) and area between the curves (Fig. 6c). Both parameters confirmed that pathway contributions to prediction rules are distributed differently for true and fake pathway sets. This supports our hypothesis that models based on true pathway sets are statistically significant: they are simpler, in that they highlight fewer important pathways. Similar results were found for the leukaemia dataset (Additional file 1: Figure S4).

### An application to investigation of 12 pathway databases

**Fig. 7:**
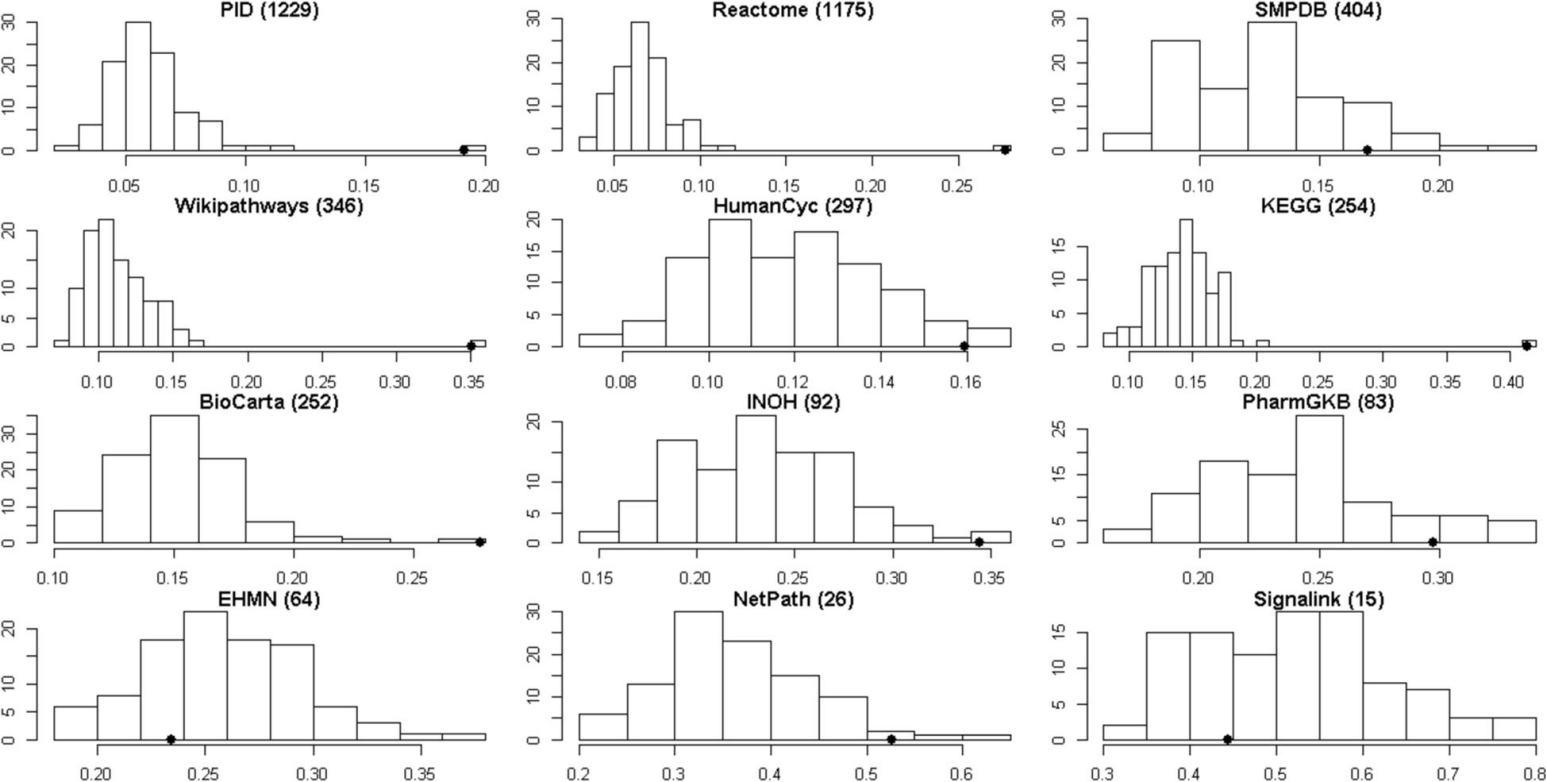
Investigation of different pathway databases. Distributions of area between curves for fake pathways (bars) versus real pathways (filled circle). The number of pathways in each database is indicated in parentheses in each panel. Results for the entropy metric can be found in Additional file 1: Figure S6.1

The large set of pathways used in this work is a compilation of many individual pathway databases. To investigate the nature of these collections, we applied the analysis of the previous section to each of the contributing databases. The results comparing real and fake pathways for each source database were summarised using the area between curves (Fig. 7). A number of databases exhibited little difference in their coefficient distributions compared to randomised collections, thus lacking significance. These included SMPDB, PharmaGKB, EMHN and Signalink. The equivalent analysis using the entropy statistic is shown in Additional file 1: Figure S6.1. At first glance, Fig. 7 appears to show a relationship between the departure from randomness and the size of databases. To investigate this, we took a large database (Reactome) and randomly subsampled it to produce smaller databases of different sizes. We found no clear relationship between database size and the departure from randomness (Additional file 1: Figure S6.2). Thus we conclude that the trends seen in Fig. 7 are not purely due to database size.

## Discussion

Evidence for the advantage of prediction based on pathways over individual genes has remained conflicting, with comparable evidence for the advantage [3] and indifference of pathway based prediction [14, 19–21]. Previous studies evaluated consistency of active pathways across data sets (e.g [5], [7], [8]) or the achievement of superior predictive accuracy (e.g. [20]), but with the exception of Holec et al., [20], all these approaches use pathway analysis as a posthoc analysis seeking to enhance the interpretability of lists of differentially expressed genes through a reduction performed with hypergeometric overrepresentation or GSEA [22]. Instead, we have built on existing approaches that utilise pathways explicitly as predictors, hence modelling in the “pathway space”. Using multivariate models in the pathway space we have devised a novel strategy to quantify the robustness of pathway collections as compared to ungrouped genes. In addition our pathway space representation revealed a potential defining characteristic of genuine pathways as opposed to random ones. It should be emphasised again, that our purpose is not to introduce a new pathway scoring method, but to use simple or existing approaches to test the hypothesis that pathway representations are more robust than those based on genes alone.

Our proposed “predictive robustness” measure assesses the ability of models to sustain predictive accuracy when the data is degraded. In this work, strong levels of perturbation to the original data resulted in lower predictive robustness of gene space models compared to that of pathway space models. Multiple controls suggested that these results are independent of the specific numerical methods or data sets used and thus the predictive robustness seems a more general property of a given pathway collection. Notably, the accuracy of pathway space models is not superior at low levels of degradation. However, this is not critical to our concept of predictive robustness, since it takes into account the maintenance of accuracy from zero to 100% degradation of the data.

To build pathway based models we adopted a bottom-up approach that included the pathway information (the gene collections) within the input data of the models. This was implemented by aggregation of the gene expression with an unsupervised method (PCA) in order to reduce the chance of over fitting, often observed in gene expression modelling [23]. Our findings of predictive robustness confirmed the advantage of aggregation with PCA over simpler options such as the arithmetic mean used previously, in terms of accuracy and robustness [3, 20]. It should be pointed out that the higher robustness of pathway space models is not a trivial outcome of the aggregation with PCA, since predictive models from pathway and genes can both benefit from the presence of redundant information amongst non-degraded genes. In addition, we note that a single principal component typically summarises a very small fraction of the total variance of gene expression a pathway (see Additional file 1: Figure S3). Thus the higher robustness of pathway models is not simply due to PCA acting as a denoising filter, since much systematic variation is also lost in higher components.

Using randomised pathway sets we showed that the accuracy and predictive robustness of the true pathways bears little or no relation to the gene membership of the pathways as dictated by current biological knowledge. This striking result provides an important insight into the use (and misuse) of predictive models for extracting information about the explanatory variables in the ‘omics’ sciences. It is often assumed that the model captures the true relationship between the measured variables and the outcome. In contrast, when models are assessed only on their prediction performance, a plausible connection between the predictors and the outcomes is not required. A mechanistic connection between the measured variables and outcomes is not guaranteed in predictive models of omics data even when machine learning methods combine efficiently information distributed across many variables [23], including even those that are only moderately correlated to the outcome. In our case, PLS-DA could detect the information from useful genes despite their reassignment and aggregation into fake pathways. This confirms that, when modelling omics data, researchers should clearly distinguish between optimising prediction performance and model interpretation.

For the second goal of quantifying the relevance of pathway definitions for multivariate models we proposed a randomisation method that creates fake mappings between genes and pathways. Across these models the contribution of the true and fake pathway sets to prediction rules, as revealed by regression coefficients, distributed differently, suggesting that true pathways are “special” collections. Specifically, the true pathway sets had significantly fewer pathways with high regression coefficients and an excess of pathways with low coefficients. These changes in coefficient distributions, summarised though the entropy and area between distribution curves, showed clear differences between different source databases (Reactome, KEGG, PIDB, etc), showing a potential application to pathway collections in general. A second dataset reproduced all the conclusions observed throughout this work. Interestingly a recent method for pathway discovery included lasso regularisation to force sparsity in predictive models of candidate pathways [7]. This supports our observation that models based on true pathways tend to be more parsimonious.

The long lasting debate about the predictive power of pathways suggests that the effect of pathways might be weak, or perhaps that the predictive accuracy might not be an adequate metric to assess the effect of pathways. From a modelling perspective, the predictive robustness is a novel approach that acknowledges the fact that real life datasets are degraded versions of the true biological signals. Our metrics based on predictive robustness and randomisation of pathway definition could offer a fresh viewpoint and help during tasks such automated pathway discovery and pathway curation.

## Conclusions

We propose the concept of predictive robustness as a new tool to assess predictive models. Predictive robustness assesses the strengths of pathways as predictors in the face of noisy data, as opposed to the outcomes of posthoc analysis of differential gene lists. We showed that pathway-based models achieve more robust predictions than gene-based models, irrespective of the data or exact workflow used. We also observed that use of real pathways does not confer a higher quality of models than randomised pathways, raising a warning regarding the interpretation of models based on predictive ability alone. However, we found that models based on true pathways are simpler, in that fewer pathways contribute strongly, when compared to those based on random collections of genes. While the current work assesses the robustness of collections of pathways, an intriguing next step in this area would be an approach which quantifies the predictive robustness of individual pathways or gene sets. This might then be used in assessing the quality of different pathway definitions.

## Methods

### Transcriptomic datasets and pre-processing

We employed two independent transcriptomic data sets. The first dataset derives from the carcinoGENOMICS (CG) project [17] intended to derive gene expression signatures that predict the carcinogenicity of compounds from in vitro cell systems (GEO accession GSE48990). The 156 samples in the data set were hybridised to Affymetrix Human Genome U133 Plus 2.0 arrays which included 60 samples treated with 6 Genotoxic compounds, 48 samples treated with 5 Non-Genotoxic compounds and 48 samples treated with 5 Non-Carcinogenic compounds. The second data set comprises microarray gene expression measurements on the Affymetrix HuGeneFL Hu6800 array of 72 samples from two leukaemia subtypes (47 ALL type and 25 AML type samples already preprocessed and available from Broad Institute website) [18]. The first data set was pre-processed using RMA normalisation with the default parameters implemented in the R package affy [24]. Pathway definitions for 4233 pathways were downloaded from the Consensus Pathway Data Base (CPDB) [25] which combines pathways from a variety of widely used source databases. We only retained those 20,307 genes which were mapped to at least one pathway within the CPDB set.

### Pathway scoring, signal degradation and degradation profiles

Our approach requires us to summarise the expression of all genes in each pathway by a score for every biological sample. Thus, gene space data matrix of *n* samples by *m* genes can be transformed to a pathway space matrix of *n* samples by *k* pathways – see Fig. 1. By default, the pathway score was taken as the score on the first principal component of the expression data of the genes in a given pathway. Alternatively, we explored the effect of using three principal components per pathway, using the mean expression of all genes in the pathway as the score (‘mean aggregation’), and using a previously published method – single sample Gene Set Enrichment Analysis (ssGSEA) [16]. By default, degradation of gene expression profiles was performed by selecting genes at random and replacing the data with values sampled from a Normal distribution with the same mean and variance as the original gene. An alternative degradation method consisted of randomly permuting the order of the samples in the expression matrix for the selected genes. The proportion of genes affected was varied from 0 to 100% to simulate different levels of noise in the data. The curve of predictive accuracy vs. level of noise (e.g. proportion of probes degraded) is termed a ‘degradation profile’ – see Fig. 2 as an example - and indicates how robust each model is to increasing levels of noise in the data. At each degradation level 20 data sets were generated with different sets of degraded genes. For every realisation of the degraded gene expression matrix, we updated the pathway score matrix accordingly, e.g. by recomputing the principal component scores.

### Predictive models and robustness statistic

Both the gene level and pathway level data were separately used to build multivariate models predictive of relevant outcomes: carcinogenicity class (3 classes) in the CG data and disease subtype (2 classes) for the leukaemia data. Models were built using Partial Least Squares-Discriminant Analysis (PLS-DA), k-Nearest Neighbour (kNN) and a linear support vector machines (SVM) and prediction accuracy measured using cross-validation. Accuracy was defined as the number of correctly classified samples divided by the total number of samples. In the case of the CG data, all samples from a given compound treatment were left out in the test set at each round. In the case of the Leukaemia data, balanced 2-fold cross-validation was used. The model complexity is given by the number of components in the case of PLS-DA, the number of neighbours in kNN and the soft threshold in SVM. The complexity was selected separately for gene and pathway models as follows: First the median predictive accuracy was computed for each complexity at each degradation level. Next the sum of the median accuracies across degradation levels was calculated for each model complexity. The optimal complexity was selected as the one which maximized this sum (see Additional file 1: Figures S5.1-S5.9). We re-estimated the required complexity of models for each variation to the workflow.

The local robustness, *r*, was quantified as the ratio of the median accuracy, $\overline{a}$ at a given level of degradation, *u,* to the accuracy on the undegraded data (*u =* 0):

1 $$ {r}_u=\overline{a_u}/{a}_0, $$

The overall predictive robustness was then defined as the area under the local robustness curve:

2 $$ R={\int}_0^1{r}_u du $$

which we evaluated using a discrete set of degradation levels {*d*_*i*_} and the trapezium rule. An approximate confidence interval for R was calculated by estimating the robustness for each of the 20 repeats and identifying the 5 and 95% percentiles of this sample. Note that the differences in predictive robustness between gene and pathway models were insensitive to the exact complexity of the models (Additional file 1: Figure S5.1-S5.9).

### Generation of fake pathway sets to assess pathway significance

The generation of fake pathway sets was performed in two ways: the default approach was to randomly permute the labels of the genes in the microarray, which preserves both the pathway sizes and the overlap of gene membership between pathways. A second approach was to create groups of randomly selected genes of the same size as the true pathways.

The distribution of PLS-DA regression coefficients in models based on true and fake pathway sets was characterised through measures of Shannon entropy and an area between curves (ABC) statistic. The entropy was calculated as

$$ H(b)=-{\sum}_{i=1}^kp\left({b}_i\right)\ln \left(p\left({b}_i\right)\right) $$

where {*b*_*i*_} are the PLS-DA regression coefficients for a given pathway collection. The probability of a given coefficient p(*b*_*i*_) was estimated using a histogram of *b*, based on 50 bins. The ABC statistic consisted of the sum of absolute areas enclosed between distributions of coefficients from each real pathway collection and the median distribution from all fake pathway sets.

All data analysis was performed with R (version 2.7) using custom written scripts and the R packages “class”,“caret” and “e1071” for kNN, PLS-DA and SVM models, respectively.

## Supplementary information

**Additional file 1.** Accompanying file with multiple figures that further support those findings described in the body of this article.

## Data Availability

The datasets analysed in the current study are available in the GEO repository https://www.ncbi.nlm.nih.gov/geo/query/acc.cgi?acc=GSE48990 and the Broad Institute public repository http://portals.broadinstitute.org/cgi-bin/cancer/publications/pub_paper.cgi?mode=view&paper_id=43.

## Abbreviations

ABC: Area Between Curves
CG: CarcinoGENOMICS
CPDB: Consensus Pathway Database
GSEA: Gene Set Enrichment Analysis
kNN: k-nearest neighbours
PCA: Principal Components Analysis
PLS-DA: Partial Least Squares – Discriminant Analysis
ssGSEA: single sample Gene Set Enrichment Analysis
SVM: Support Vector Machine
