# Supplementary material for: Predictive modelling using pathway scores: robustness and significance of pathway collections
Source: BMC Bioinformatics. 2019 Nov 4;20:543. doi: 10.1186/s12859-019-3163-0 (PMC6827178; doi:10.1186/s12859-019-3163-0)
Supplement: Supplementary file 1 — Additional file 1. Accompanying file with multiple figures that further support those findings described in the body of this article. [file 12859_2019_3163_MOESM1_ESM.docx]

**Predictive Modelling Using Pathway Scores: Robustness and Significance of Pathway Collections**

### Marcelo P. Segura-Lepe^1^, Hector C.Keun^1^ and Timothy M.D. Ebbels^1§^

^1^Computational and Systems Medicine, Department of Surgery and Cancer, Sir Alexander Fleming building, Imperial College, London, SW1 2AZ, UK.

# Additional file

## Table S1- Predictive robustness for the various combinations of workflow steps and data sets

Empirical 90% confidence intervals were calculated for predictive robustness using 20 repeats at each level of degradation. The models with highest predictive robustness are indicated in bold.

|  | Predictive Robustness % (Equation 2) | | | | | |
| --- | --- | --- | --- | --- | --- | --- |
| Workflow or data changes | Gene models | | | Pathway models | | |
|  | Median | 90% CI | | Median | 90% CI | |
| Basic profile (Fig.2) | 82 | 81 | 83 | **90** | **89** | **91** |
| Random permutation degradation (Fig.3A) | 81 | 81 | 82 | **87** | **86** | **88** |
| kNN (Fig.3B) | 86 | 85 | 87 | **93** | **92** | **94** |
| Leukaemia data (Fig.4) | 86 | 86 | 87 | **88** | **87** | **89** |
| “Global” Noise Scheme (Fig. S1.1)* | 5.0 | 4.9 | 5.1 | **6.0** | **5.8** | **6.1** |
| Three components per pathway (Fig. S1.2A) | 82 | 81 | 83 | **89** | **87** | **90** |
| Mean aggregation (Fig.S1.2B) | **82** | **81** | **83** | 77 | 74 | 80 |
| ssGSEA pathway scores (Fig S1.2C) | 73 | 69 | 78 | **78** | **79** | **81** |
| SVM (Fig. S1.3) | 76 | 75 | 76 | **88** | **87** | **89** |
| Gene selection (Fig. S1.4) | 87 | 84 | 89 | 87 | 84 | 92 |

* For the Global noise scheme the degradation levels have a scale based on multiples of the standard deviation, and not on the percentage of degraded genes used in all other conditions. For example, a predictive robustness of 5.0 indicates that the accuracy drops 50% of its maximal value when the added noise is five times the standard deviation of the unperturbed data.

| 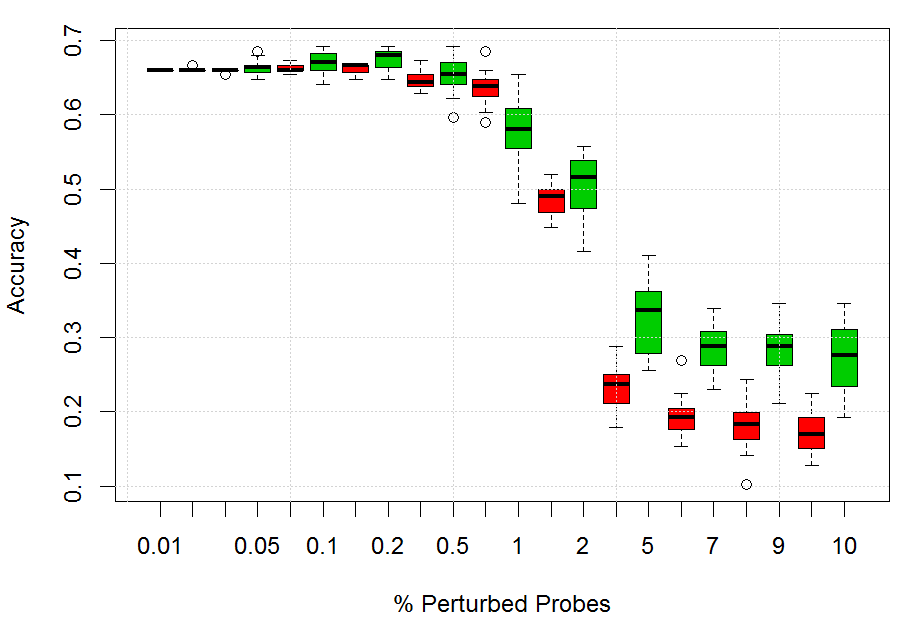  Level of added Gaussian noise multiplication factor  **C** |
| --- |

## Figure S1.1 - Modifications to the workflow: global noise addition

The influence on the degradation profile of pathway scores was calculated using a “global noise” strategy in which Gaussian noise was added to each gene with variance a constant fraction of the original gene expression variance.

| 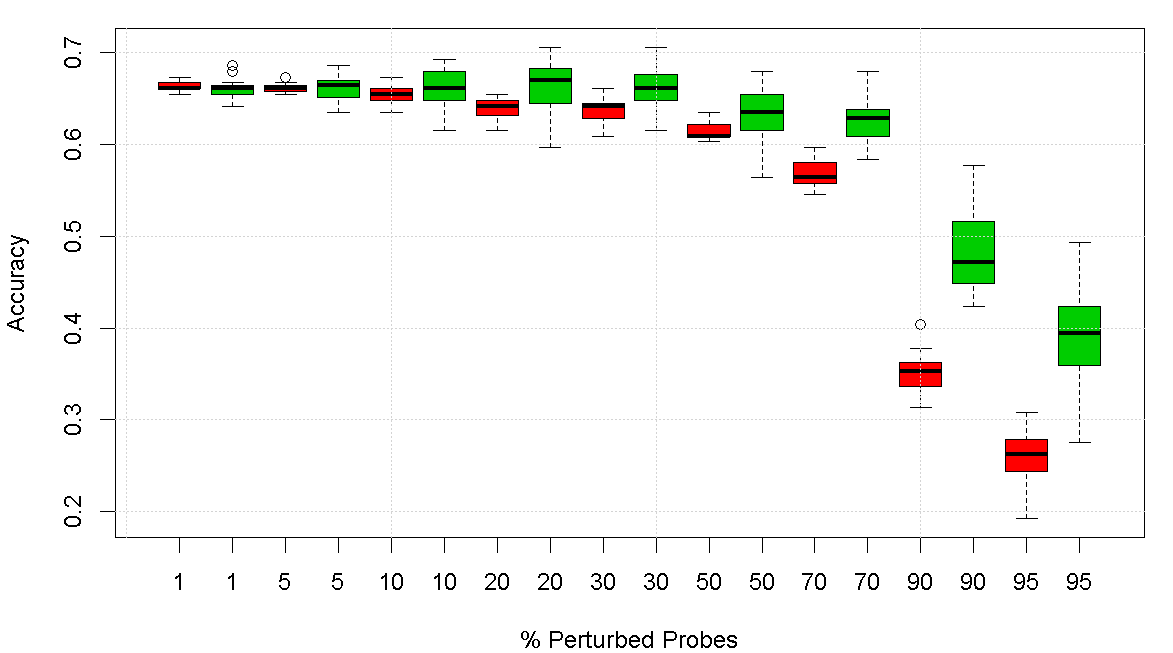  **A** | 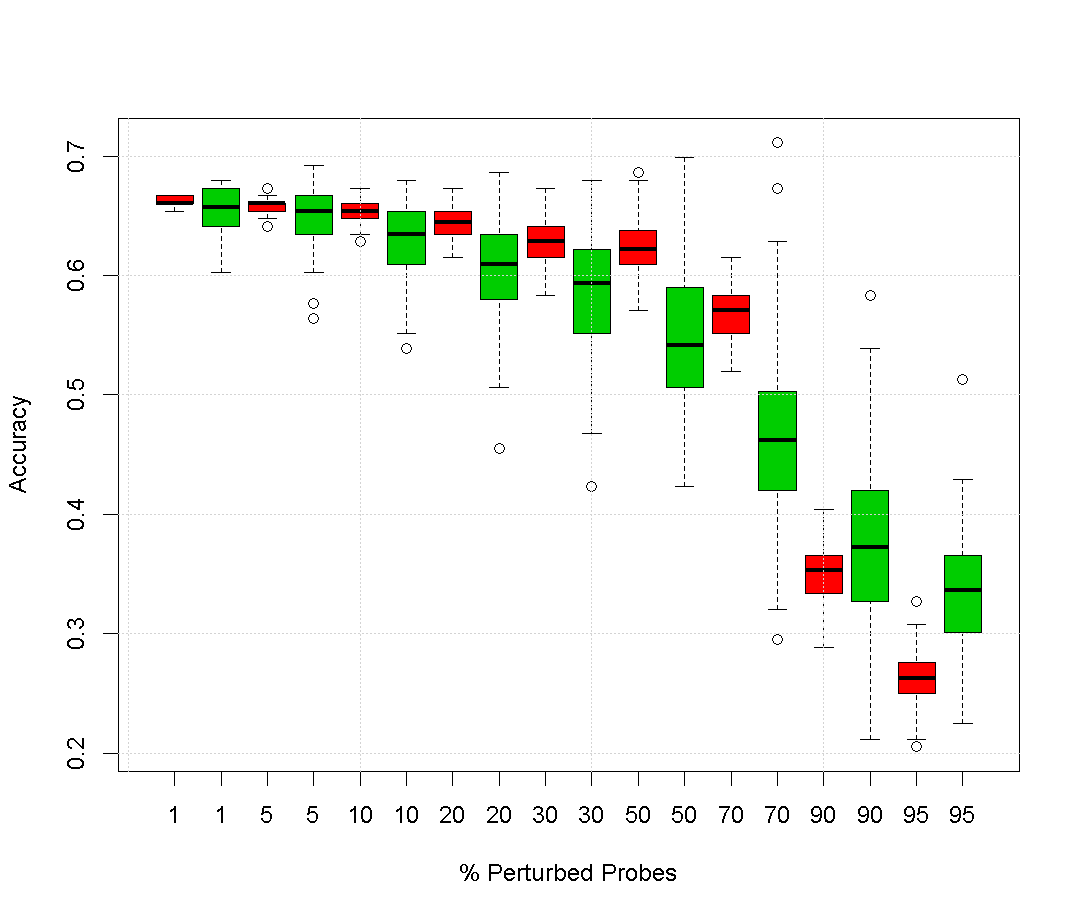  **B** |
| --- | --- |
| 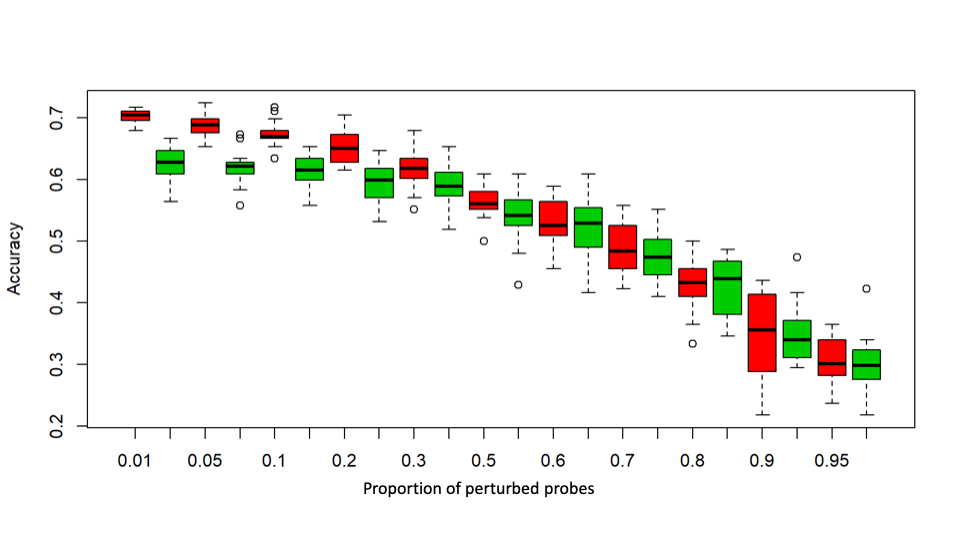  **C** | |

## Figure S1.2 - Modifications to the workflow: Pathway scoring using three principal components, mean aggregation and ssGSEA.

The influence on the degradation profile of pathway scores was calculated using either 3 principal components per pathway (A), aggregation of genes based on arithmetic mean (B), or using ssGSEA (C). All modifications were tested while maintaining all the other steps of the workflow as applied in Figure 2 (and for the CG data set).


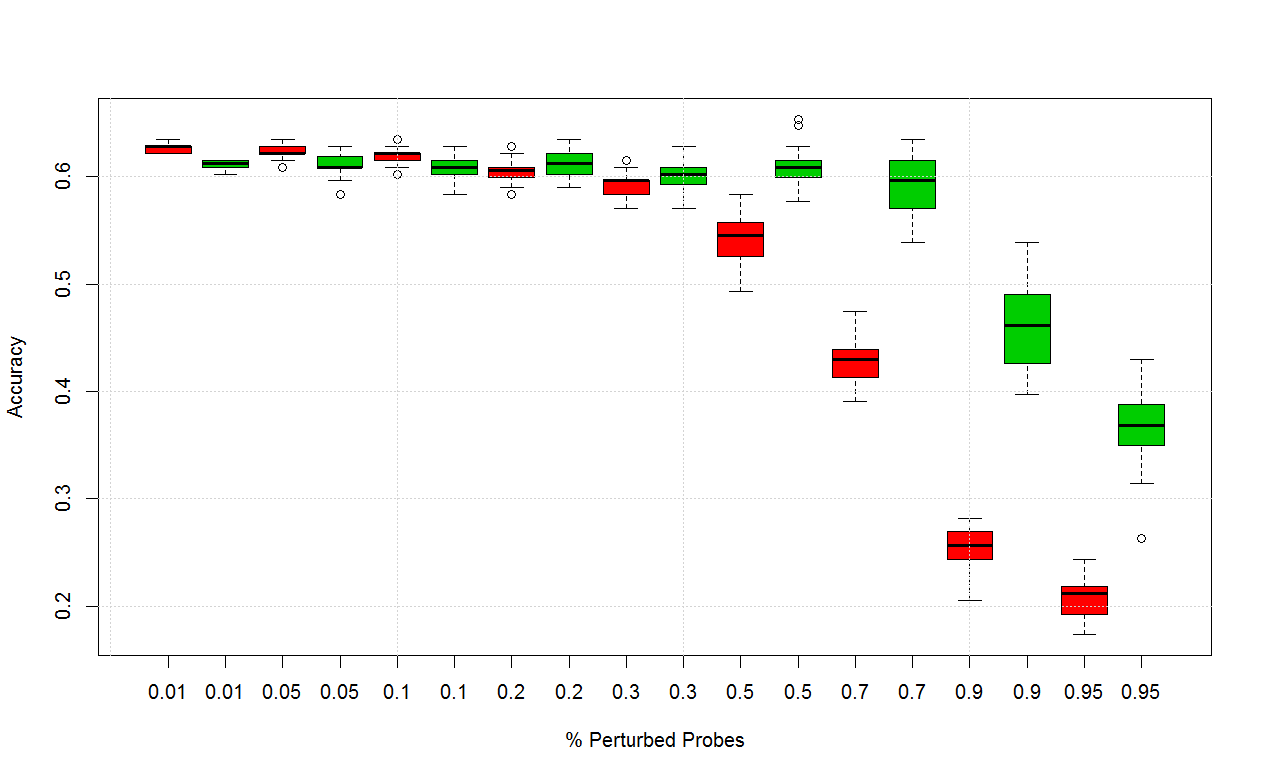


## Figure S1.3 Modifications to the workflow: SVM classifier

The influence on the degradation profile of pathway scores was investigated using SVM as classifier. A linear kernel was used as it produced more accurate predictions compared to others (e.g. RBF). The additional classifier was trained in a similar fashion to PLS-DA and KNN, using a grid search to find optimal model parameters. In the case of linear SVM this corresponds to the single parameter for the soft threshold.


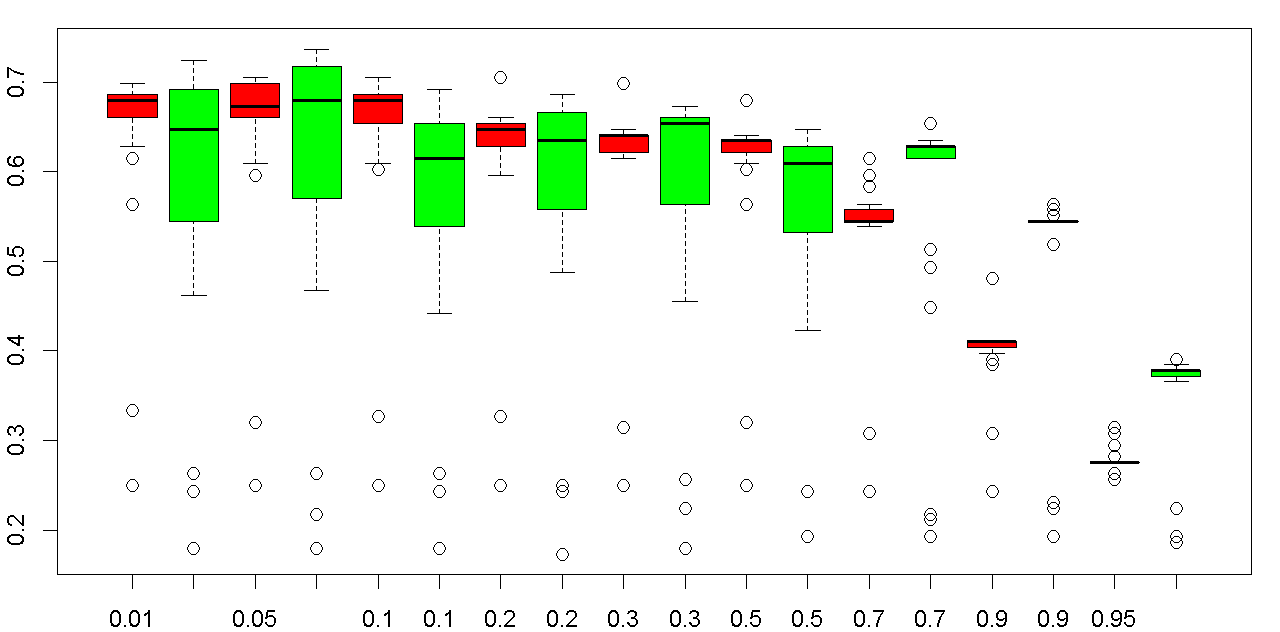


## Figure S1.4 - Modifications to the workflow: gene selection

The influence on the degradation profile of genes under feature selection was investigated for the CG data set. Gene models were built using only genes found to be differentially expressed after degradation (red series). In each case the top 500 differentially expressed genes were identified by ANOVA Construction of pathway models remained unaltered, using all genes available to each pathway (green series).


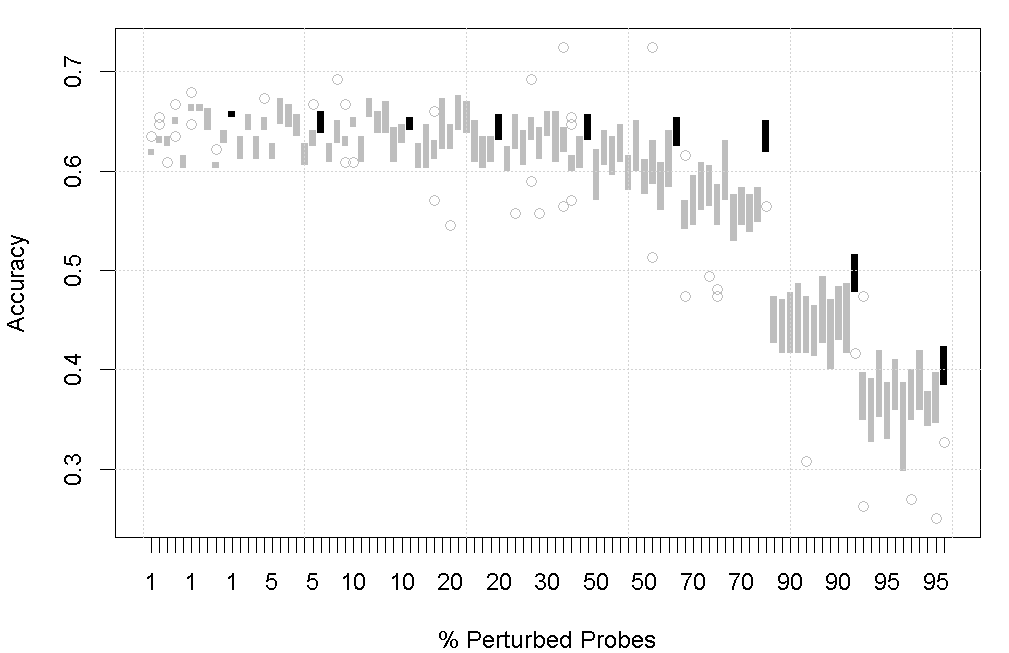


## Figure S2. Degradation profiles for true and fake pathway sets with alternative randomisation scheme

The alternative scheme preserves the size of the original pathways without constraints on the overlap of genes across pathways. The CG dataset was used.


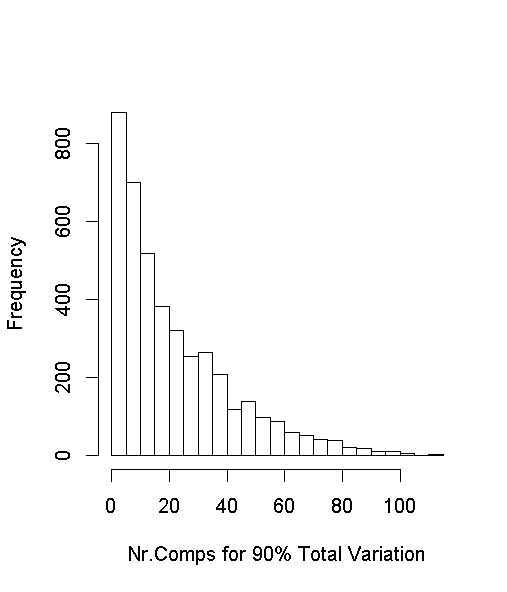


## Figure S3. Distribution of the number of principal components required to capture 90% of gene expression variance in the pathway. 25, 50 and 75^th^ percentiles of the distribution are found at 7, 16 & 33 components.

| Accuracy | 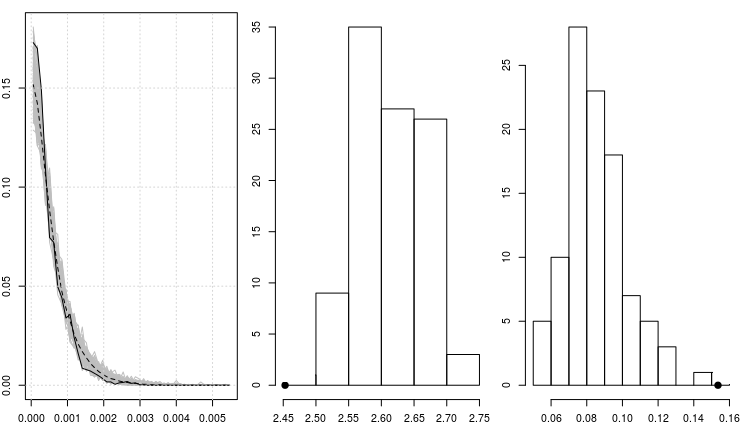 | | |
| --- | --- | --- | --- |
|  | PLSDA Coefficients (absolute) | Entropy | Area between Curves |

## Figure S4 - Distributions of PLS-DA regression coefficients, entropy and area between the curves for the leukaemia data set

The solid black line and filled circles indicate the true pathway set.

# Accuracy of models with different complexities

For all the following figures the cross validated accuracy across all degradation levels and repeats is plotted against the complexity of the model (grey curves). The red curves correspond to the median accuracy for all the repeats of the same degradation level. The selected complexity (vertical black line in each plot) maximises the sum of median accuracies achieved by a given complexity across all degradation levels. Note the consistency of the degradation profile in both PLS-DA and kNN models, indicating a lack of sensitivity to the exact model complexity.

| Accuracy | 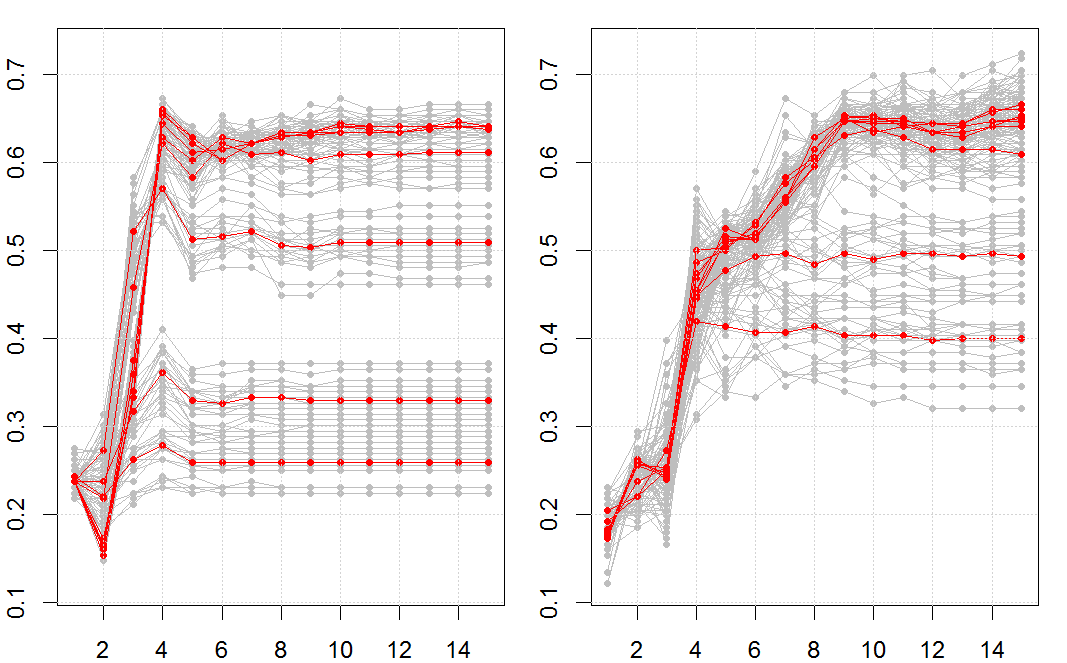 | |
| --- | --- | --- |
|  | Nr. Components ( Gene model complexity) | Nr. Components (Pathway model complexity) |

## Figure S5.1 - Selection of complexity for models calculated using the default workflow (See Fig.2)

| Accuracy | 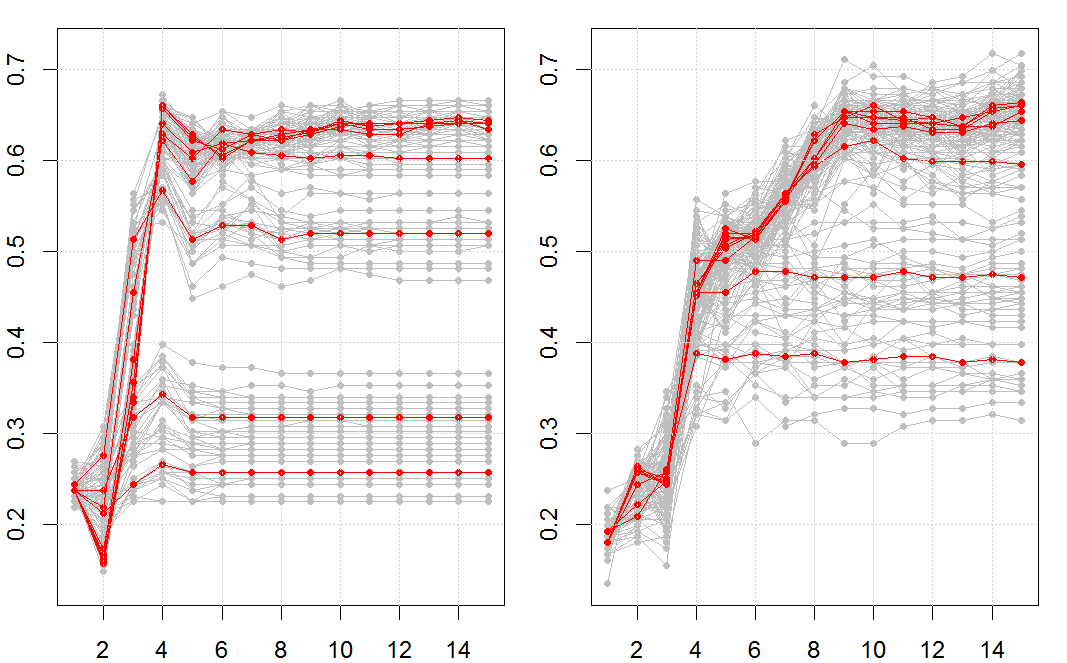 | |
| --- | --- | --- |
|  | Nr. Components ( Gene model complexity) | Nr. Components (Pathway model complexity) |

## Figure S5.2 - Selection of complexity for models for modified workflow: Random permutation to degrade genes (See Fig.3A)

| Accuracy | 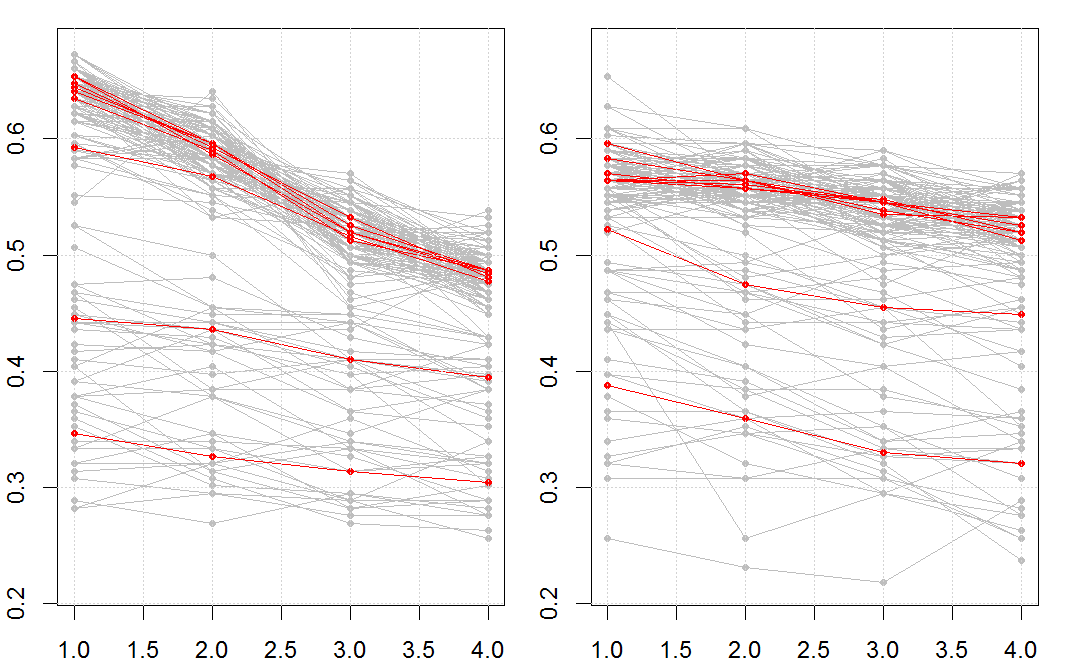 | |
| --- | --- | --- |
|  | Nr. Neighbours ( Gene model complexity) | Nr. Neighbours (Pathway model complexity) |

## Figure S5.3 - Selection of complexity for models for modified workflow: kNN based classification (See Fig.3B)

| Accuracy | 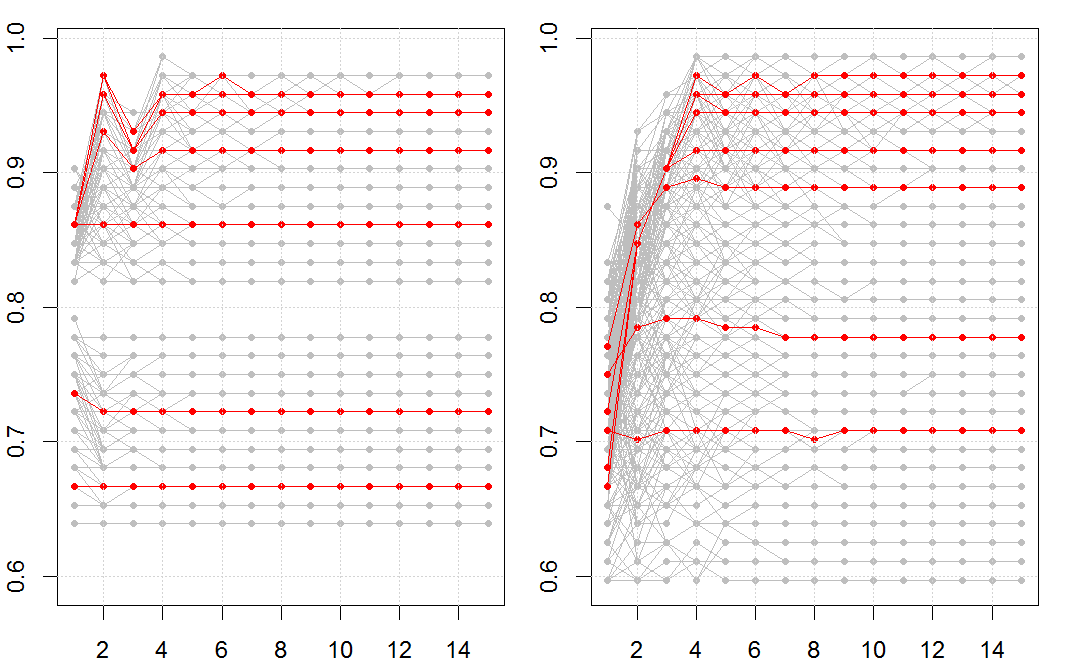 | |
| --- | --- | --- |
|  | Nr. Components ( Gene model complexity) | Nr. Components (Pathway model complexity) |

## Figure S5.4 - Selection of complexity for modified workflow: Initial dataset replaced by leukaemia dataset (See Fig.4)

| Accuracy | 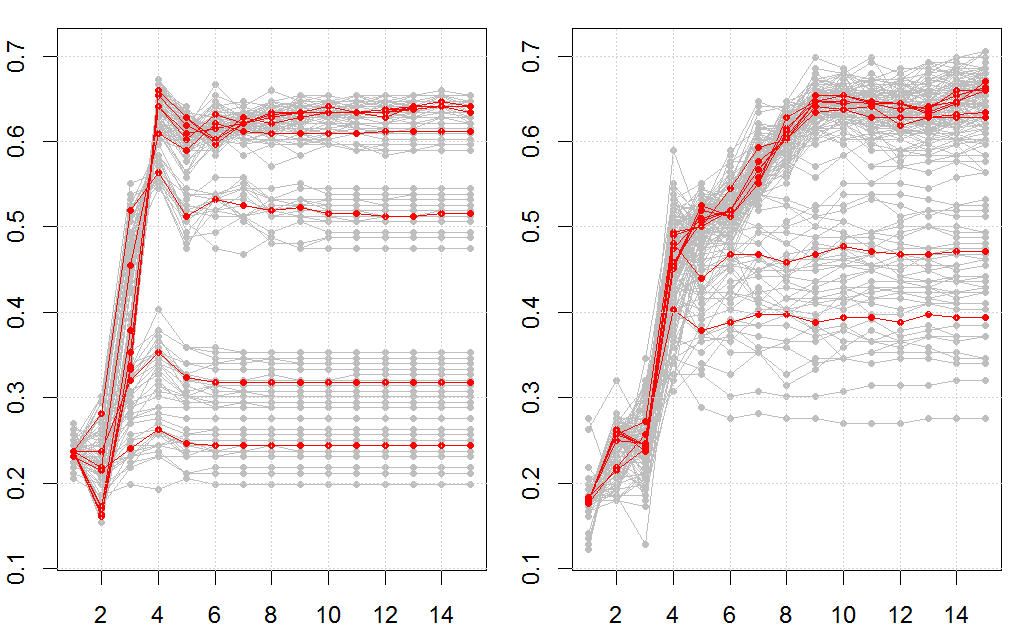 | |
| --- | --- | --- |
|  | Nr. Components ( Gene model complexity) | Nr. Components (Pathway model complexity) |

## Figure S5.5 - Selection of model complexity for modified workflow: pathway space based on 3 principal components per pathway (See Fig.S1.2A)

| Accuracy | 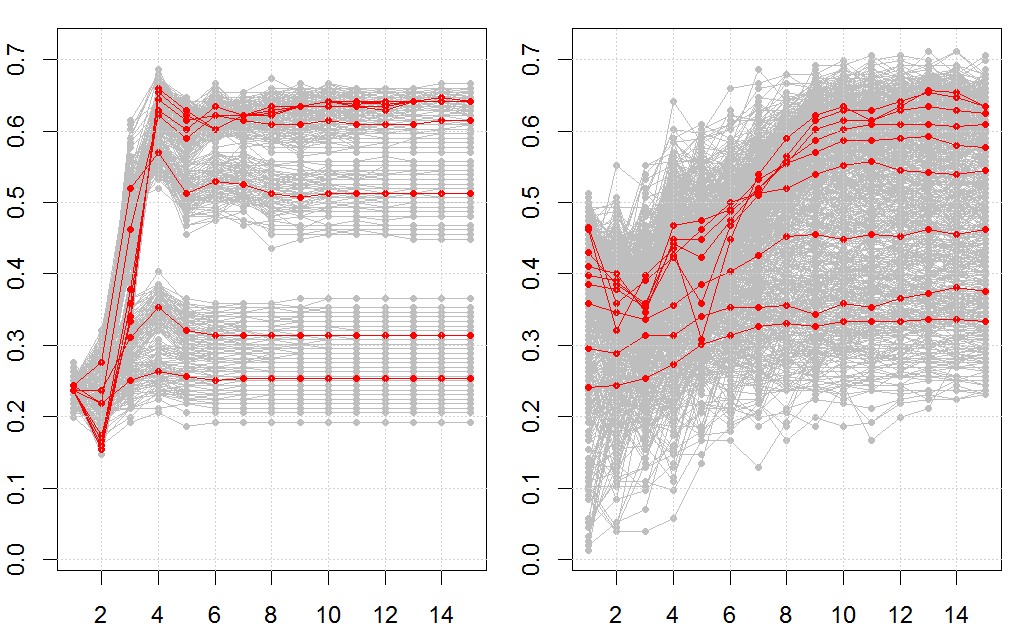 | |
| --- | --- | --- |
|  | Nr. Components ( Gene model complexity) | Nr. Components (Pathway model complexity) |

## Figure S5.6 -. Selection of model complexity for modified workflow: Aggregation of pathways based on arithmetic mean (Fig. S1.2B)

| Accuracy | 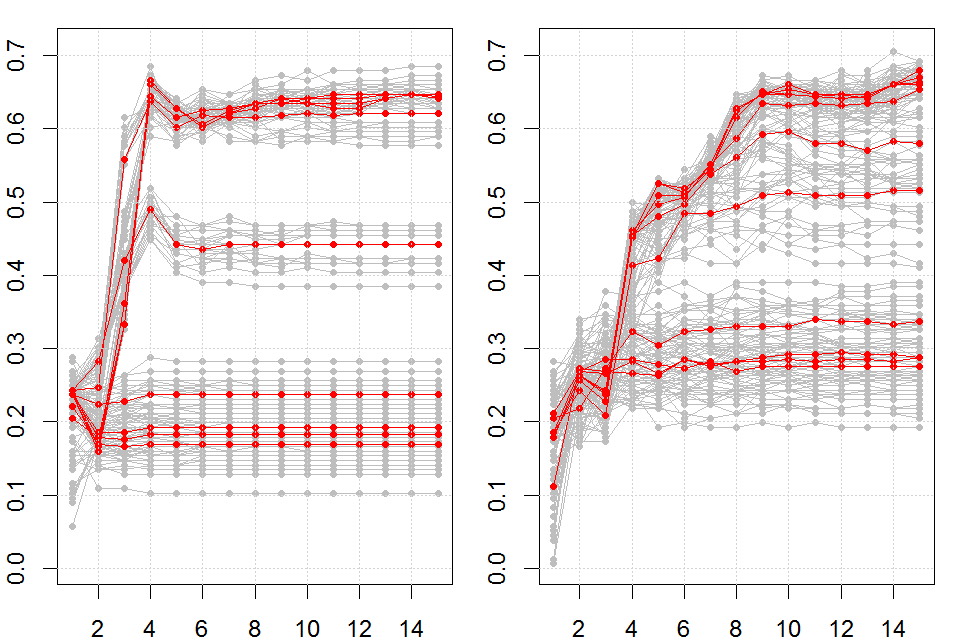 | |
| --- | --- | --- |
|  | Nr. Components ( Gene model complexity) | Nr. Components (Pathway model complexity) |

## Figure S5.7 - Selection of model complexity for modified workflow: alternative “Global” noise scheme (Fig. S1.1)

| Accuracy | 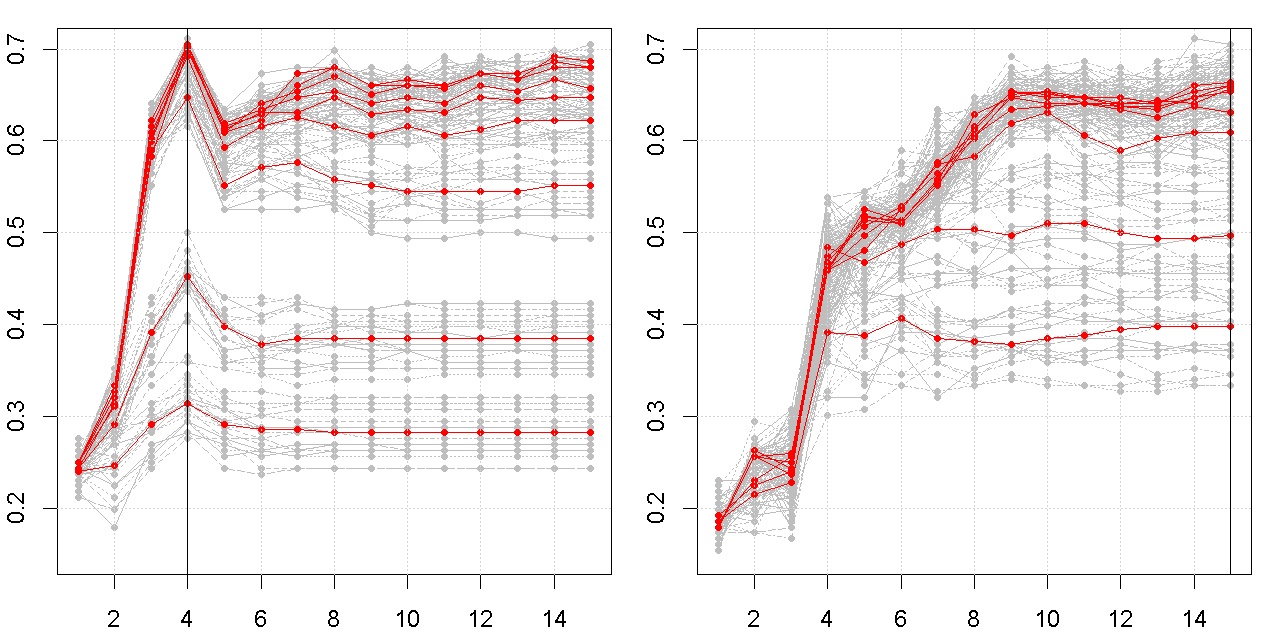 | |
| --- | --- | --- |
|  | Nr. Components ( Gene model complexity) | Nr. Components (Pathway model complexity) |

## Figure S5.8 - Selection of model complexity for modified workflow: alternative based on gene selection (Fig S1.4)


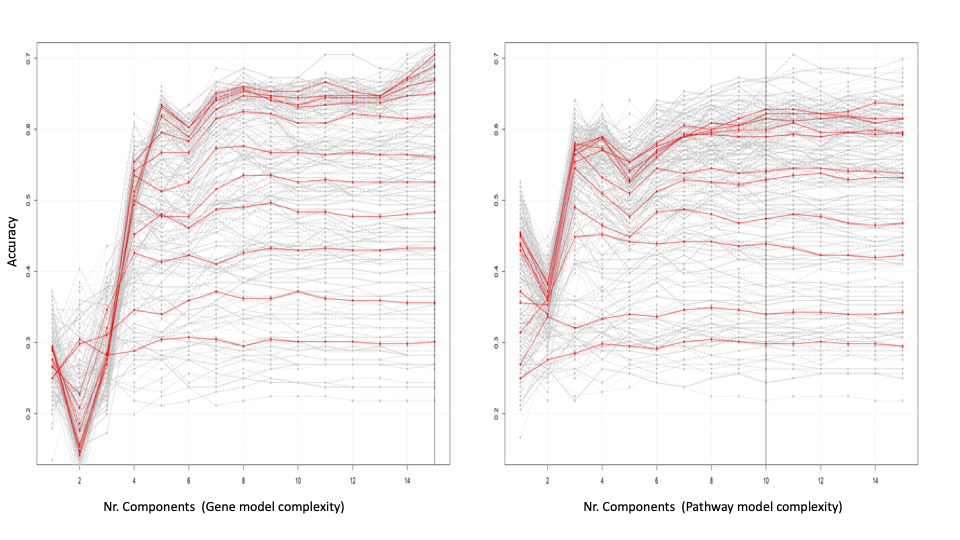


## Figure S5.9 - Selection of model complexity for modified workflow: alternative based on ssGSEA pathway scores (Fig S1.3C)


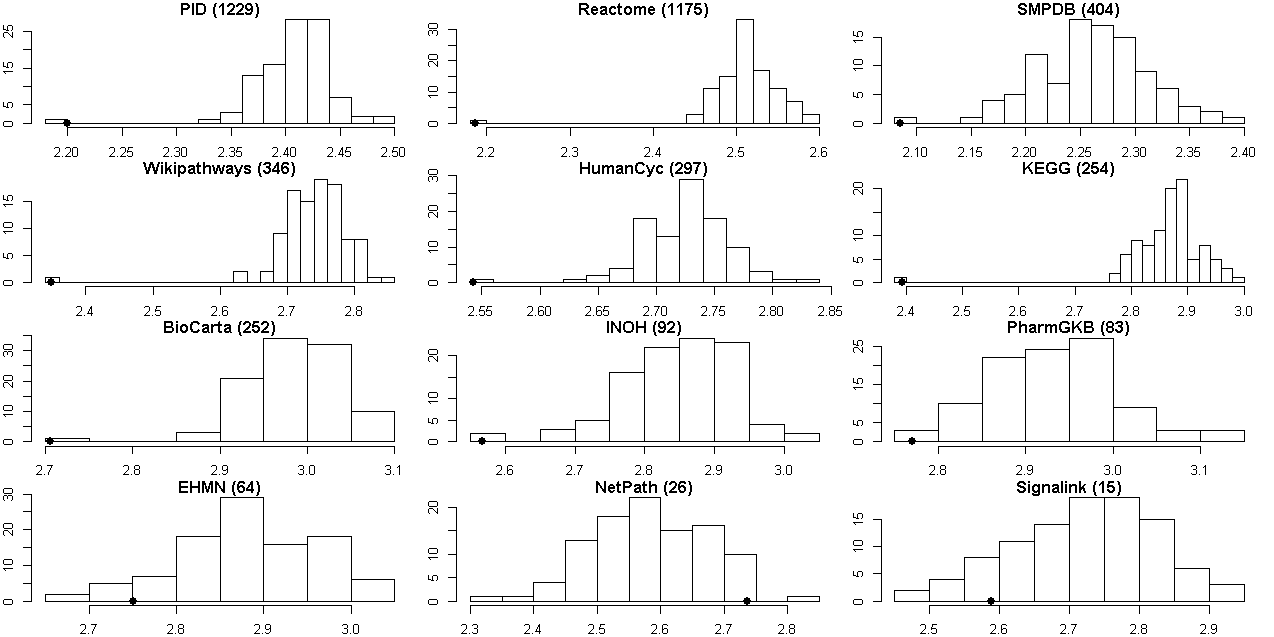


## Figure S6.1 – Entropy of the regression coefficients from PLSDA models from true and randomised pathways for different databases. In each case the histogram gives the distribution of entropies measured on randomised pathways. The solid dot gives the entropy for the true pathway definitions.


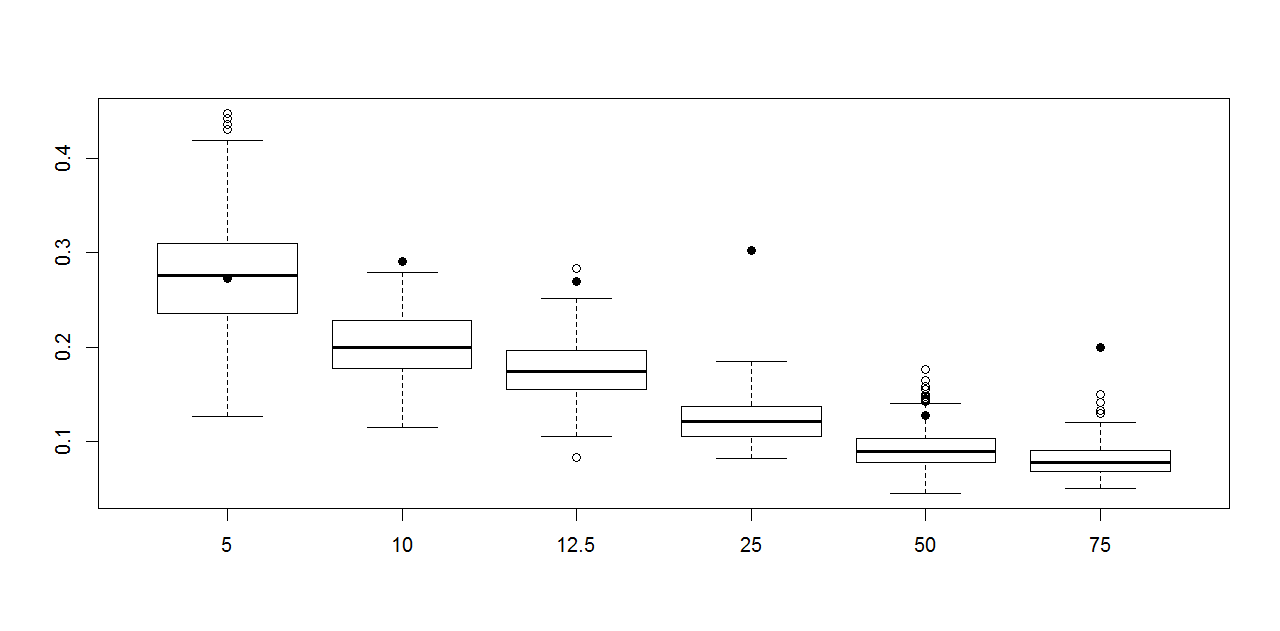


## Figure S6.2 – Effect of database size on the distribution of prediction rule coefficients. Using Reactome as an example, we created databases of different sizes by selecting pathways at random without replacement. For each sub-database, 100 versions with randomised pathways were created. PLSDA models were computed for each sub-database (true and randomized). As in the main text, the difference in the distributions of PLSDA coefficients between true and mean random versions of the subsampled pathway set were summarised through the area between distribution curves (as for Figure 6C). The box plots give the distribution of these enclosed areas for each database size. In each plot, the solid circle represents the true pathway definitions. The departure from randomness is taken as the distance of the solid circle from the median compared to the distribution width (size of the box).

Area between curves (deviation from median) distribution of PLSDA coefficients)

% Reactome pathways subsampled
